# Air Subdivision Research of Laser Atmospheric Propagation Between Dual Reflectors of the Large-Aperture Antenna

**DOI:** 10.3390/s26041207

**Published:** 2026-02-12

**Authors:** Xuan Zhang, Xijie Li, Hu Wang, Ming Gao, Yunqiang Lai, Hong Lv

**Affiliations:** 1 School of Opto-Electronical Engineering, Xi’an Technological University, No.2 Xuefuzhonglu Road, Xi’an 710021, China; 2 Shaanxi Key Laboratory of Optical Remote Sensing and Intelligent Information Processing, Xi’an Institute of Optics and Precision Mechanics, Chinese Academy of Sciences, Xi’an 710119, China; 3 State Key Laboratory of Ultrafast Optical Science and Technology, Xi’an Institute of Optics and Precision Mechanics, CAS, Xi’an 710119, China; 4 University of Chinese Academy of Sciences, Beijing 101408, China; 5 Xi’an Space Sensor Optical Technology Engineering Research Centre, Xi’an 710019, China

**Keywords:** inhomogeneous atmospheric subdivision, dual reflector surfaces, temperature gradients, coupled thermal fluid dynamics, laser atmospheric propagation

## Abstract

Laser measurement technology is widely used for deformation or pose monitoring of the dual-reflector antenna systems. However, conventional models of surface temperature variation with altitude fail to accurately characterise the temperature gradients between the main reflector and the subreflector of the large-aperture antennas, due to the complex near-ground environment, the antenna’s dual-reflector structural properties, and the antenna’s own rotation changes. This temperature modelling discrepancy significantly influences the laser atmospheric propagation deflection characteristics, ultimately leading to a decrease in the accuracy of antenna attitude measurements. To address these issues, this paper proposes a theory of air stratification within large-aperture antennas and utilizes this theory to optimize the temperature gradient between the antenna’s dual reflectors. Secondly, a coupled heat-fluid dynamics model for the dual-reflector surfaces is established using Computational Fluid Dynamics to simulate the atmospheric stratification under different rotational positions of the antenna. Finally, the effectiveness and feasibility of the proposed theory were verified through experiments in the antenna model and the China Nanshan 25 m non-rotatable antenna. This research provides an original theoretical and practical basis for precision environmental modelling in antenna measurements, offering prior assurance for improving the accuracy of laser-based antenna attitude measurement.

## 1. Introduction

With the rapid development of China’s space industry and astronomical research, large-aperture radio telescope antennas are playing an increasingly important role in deep space exploration. Large-aperture reflector antennas are primarily used for astronomical observations such as gravitational waves, radio bursts, cosmology, astrochemistry, black holes, and dark matter, etc. [1]. Therefore, the accuracy of antenna observations is crucial, and precise pose or surface shape measurement between the main reflector and subreflector of the antenna is a key factor in ensuring antenna performance indicators. However, conventional temperature height-dependent models—such as the International Standard Atmosphere (ISA) model with its characteristic lapse rate of −6.5 °C/km [2]—fail to accurately characterise the temperature gradients between the main reflector and subreflector of the large-aperture antennas. This inaccuracy arises due to the complex near-ground environment, the structural characteristics of the antenna’s dual reflection surfaces, and the antenna motion pose changes. It is particularly noteworthy that the main reflector not only generates thermal radiation due to its own heating, but its reflection surface structure is more likely to concentrate thermal energy (including solar radiation and its own thermal radiation) in the area of the subreflector, resulting in an abnormal increase in the temperature of the subreflector. The environment between the dual reflector surfaces of the antenna is also disturbed by atmospheric turbulence, essentially forming an atmospheric layered structure with significant vertical gradients. This complex temperature stratification directly leads to the laser atmospheric propagation deflection. It should be noted that the temperature difference between the main reflector and subreflector of the antenna is significantly influenced by external factors such as season, time, weather conditions, and wind speed. However, the primary factor affecting temperature gradient changes is airflow disturbance. The temperature model deviation mentioned significantly affects the deflection characteristics of laser atmospheric propagation, ultimately leading to a decrease in the accuracy of high-precision antenna pose measurements, which in turn affects the observation accuracy of large aperture antennas [3]. To solve these problems, it is necessary to fully understand the principle of air stratification between the main and secondary surfaces of large aperture antennas. The precise monitoring and optimization of the environment between the dual reflector surfaces of large-aperture antennas must be based on an accurate understanding of their layered structure, due to complex factors such as electromagnetic shielding requirements, attitude adjustment during operation, as well as solar thermal effects and asymmetric airflow characteristics induced by the dual-reflector configuration. Based on this, investigating the laser propagation deflection caused by inhomogeneous air stratification between the dual reflectors holds significant theoretical value for improving the observational performance of the antenna systems.

The study of atmospheric subdivision is extremely important in atmospheric science and involves various types, including turbulent stratification, thermal stratification, stability stratification, density stratification, and radiative stratification [4,5,6,7,8,9]. Numerous studies have shown that surface temperature varies with altitude following certain functional relationships [10,11,12]. Kim conducted a parametric study on the stability of the air layer [13], Liang established a calculation model for the atmospheric stratification transmittance of satellite channels through machine learning [14], and Xu et al. discussed the near-ground air stratification model and derived the atmospheric refraction correction formula [15]. The influence mechanism of wind and atmospheric stratification has also been investigated [8,16]. Zhang studied the non-uniform atmospheric refraction model in near space [17], and Wang et al. examined the light propagation performance under non-uniform atmospheric smoke [18]. Additionally, some scholars have focused on atmospheric refraction effects and error analysis [19,20,21]. These studies collectively provide a theoretical foundation for the research on inhomogeneous atmospheric stratification presented in this paper.

Regarding the research on the temperature field of radio telescope antennas, Wei et al. studied the non-uniform temperature field of the main reflector of a large radio telescope [22]. Other studies have presented analyses of radiant temperature variation and error analysis for the main reflector and subreflector [23,24,25,26]. Yi et al. studied the temperature field of the antenna structure of the Nanshan 25 m radio telescope antenna, showing that the temperature distribution was approximately linear [27]. While numerous studies have focused on the thermal radiation or temperature gradient variations in the main reflector and the backing structure of radio telescope antennas [22,23,24,25,26,27,28], no relevant articles addressing precise temperature stratification and gradient variations in the region between the main reflector and the subreflector have been found to date. Furthermore, environmental temperature assessments of antennas mainly rely on meteorological stations near the antenna [27,29,30]. However, temperature variation across a geographical area differs significantly from the temperature gradient between the antenna’s main reflector and sub-reflector and cannot directly reflect the real air stratification between them. Therefore, it is necessary to study the inhomogeneous air layering phenomenon in the region between the dual reflectors of the large-aperture antenna.

In 1989, Zhang et al. verified that a refractive index gradient induced by a temperature gradient can cause laser propagation deflection [31]. Subsequently, other researchers studied a polynomial fitting atmospheric refraction model to improve the collimation efficiency of laser atmospheric transmission [32]. Vedad F. demonstrated through experiments with bent smoke layers that spatially varying refractive indices affect the direction and position of light propagation [33]. Furthermore, many researchers modelled the turbulence in a laser propagation path as an infinite series of isotropic layers, normally represented as phase screens [34,35,36,37,38], to study laser spot variation through atmospheric perturbations. However, these studies primarily concern long-distance laser propagation.

These studies on laser atmospheric propagation provide a theoretical basis for calculating the laser atmospheric subdivision propagation deflection between the dual-reflector antenna in this paper. This paper focuses on the phenomenon of laser propagation deflection caused by inhomogeneous air stratification between the dual reflectors of an antenna. By establishing a quantitative theoretical model that links this stratification to laser deflection, it first reveals the underlying mechanisms of the “optical environment black box” between the main reflector and subreflector surfaces of the antenna. This research not only provides the theoretical foundation for achieving high-precision attitude and position compensation but also holds critical significance for enhancing the observational performance of the entire antenna system. The main contributions in this work are threefold:

(1) A theory concerning the air stratification and laser atmospheric propagation deflection subdivision within large-aperture antennas has been proposed, and this theory has been utilised to optimize the temperature gradient between the antenna’s dual reflectors.

(2) The coupled heat-fluid dynamics model is established through Computational Fluid Dynamics (CFD) to simulate both the asymmetric airflow characteristics and the atmospheric stratification between the main reflector and subreflector of the large-aperture antenna under different working conditions.

(3) The effectiveness and feasibility of the environmental monitoring scheme of inhomogeneous atmospheric subdivision are verified by antenna model and Nanshan 25 m non-rotatable radio telescope antenna temperature measurement experiments.

In Section 2, we describe the mathematical model of the laser atmospheric propagation deflection subdivision. Section 3 presents the detailed simulation and experiments, and the data analysis results. Section 4 concludes this work.

## 2. Materials and Methods

**Figure 1:**
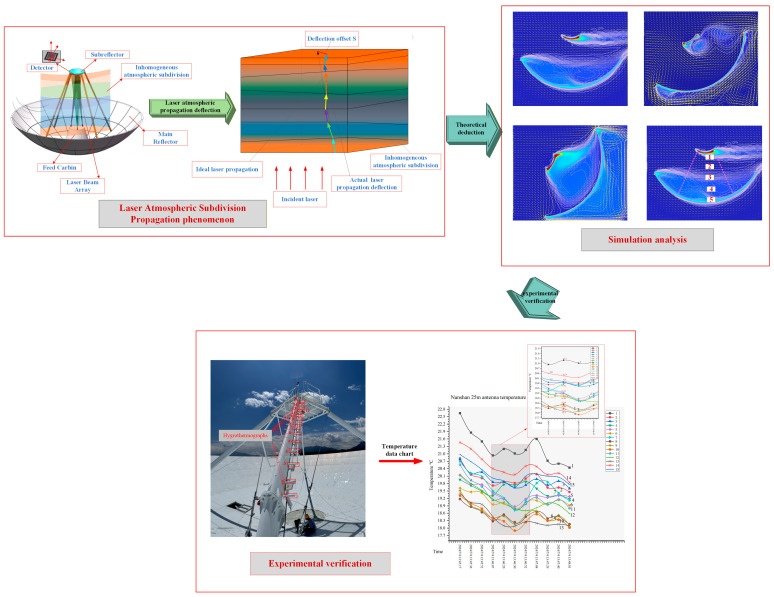
The schematic diagram of the laser atmospheric subdivision propagation.

This paper investigates the inhomogeneous atmospheric layers between the main reflector and subreflector of a large-aperture antenna. These layers are subdivided into multiple isotropic air layers. The theoretical deflection of laser beam propagation through these layers and the corresponding atmospheric radiative thickness are calculated. The effectiveness and feasibility of this approach are verified through simulation and experiment. A schematic diagram of the air subdivision for laser atmospheric propagation is presented in Figure 1.

### 2.1. Temperature Gradient Model

According to the First Law of Thermodynamics and static equilibrium equations, the temperature in the troposphere decreases by approximately 0.6 °C per 100 m increase in altitude in the absence of external thermal disturbances. However, under direct solar radiation, the temperature of the main reflector rises, while the subreflector becomes hotter than the main reflector due to energy convergence from the latter. This results in a non-uniform temperature gradient between the two reflectors at thermal equilibrium, which deviates from the conventional temperature variation with altitude. More critically, the laser deflection induced by this temperature gradient must be taken into account

The variation characteristics of the atmospheric refractive index field directly lead to laser deflection for near-ground large-aperture antenna measurement. In this paper, the Rüeger formula [39] is used to calculate the atmospheric refractive index. It is well known that laser parameters, such as wavelength and output power, can affect measurement accuracy. The wavelength influences the refractive index through dispersion, while the output power determines the signal-to-noise ratio of the detector. However, the selection of these hardware parameters is limited by irradiance, camera recognition accuracy, and practical measurement convenience. Since the main focus of this paper is on the environmental stratification mechanism between reflectors rather than hardware optimization, these laser parameters are treated as fixed control variables in this study. Based on the meteorological parameters of monitoring points, the atmospheric refractive index Ni can be calculated for each monitoring point, as shown in Equation (1).

(1) $$N_{i}=77.6890\frac{P_{i}}{K_{i}}+71.2952\times Q_{i}\frac{e_{0i}}{K_{i}}+375463\times Q_{i}\times \frac{e_{0i}}{K_{i}^{2}}$$

In Equation (1), $P_{i}$ is the pressure at the i th monitoring point, $K_{i}$ is the Kelvin temperature at the i th monitoring point, K=T+273.15, and $e_{0i}$ is the saturated water vapour pressure at the i th monitoring point. According to Equation (1), the factors that affect the refractive index are temperature, pressure, and humidity. However, the local pressure and humidity around the antenna are relatively stable, so the primary factor to consider is the temperature gradient.

The refractive gradient can be obtained by measuring the refractive index of any point in three-dimensional space, and the formula is shown in Equation (2):

(2) $$\nabla n=\frac{\partial n}{\partial x}\vec{i}+\frac{\partial n}{\partial y}\vec{j}+\frac{\partial n}{\partial z}\vec{k}$$

The refractive index of any point in the spatial flow field is a three-dimensional coordinate function, which can be expressed as: $n=n\left(x,y,z\right)$. According to the Fermat principle of integrating spatial variables, the infinitesimal arc length ds can be expressed as Equation (3):

(3) $$ds={\left[{\left(dx\right)}^{2}+{\left(dy\right)}^{2}\right]}^{1/2}={\left[1+x^{2}+y^{2}\right]}^{1/2}dz$$

According to the Lagrange equation, the relationship between spatial refractive gradient and *ds* can be followed by Equation (4):

(4) $$\left\{\begin{array}{c} \frac{d}{ds}\left(n\frac{dx}{ds}\right)=\frac{\partial n}{\partial x}\\ \frac{d}{ds}\left(n\frac{dy}{ds}\right)=\frac{\partial n}{\partial y}\\ \frac{d}{ds}\left(n\frac{dz}{ds}\right)=\frac{\partial n}{\partial z} \end{array}\right.\Rightarrow \frac{d}{ds}\left(n\frac{d\vec{r}}{ds}\right)=\nabla n$$

In Equation (4), *s* is the arc length of the laser propagation trajectory in a three-dimensional non-uniform flow field, and $r\left(x,y,z\right)$ is the spatial position coordinate of a point, where *n* and ∇n are the refractive index and its gradient, respectively.

The temperature gradient induces a spatially varying refractive index $n\left(r\right)$, which can be expressed as in Equation (5):

(5) $$n\left(r\right)=n_{0}+\frac{dn}{dT}\cdot \Delta T\left(r\right)$$

In Equation (5), $n_{0}$ is the initial refractive index, dn/dT is the thermo-optic coefficient, defined as the rate of refractive index variation per unit temperature change, $\Delta T\left(r\right)$ is defined as the spatial temperature distribution function. The relationship between the temperature gradient and the refractive index gradient can be expressed as:

(6) $$\left\{\begin{array}{c} \frac{\partial n}{\partial x}=\frac{dn}{dT}\cdot \frac{\partial T}{\partial x}\\ \frac{\partial n}{\partial y}=\frac{dn}{dT}\cdot \frac{\partial T}{\partial y}\\ \frac{\partial n}{\partial z}=\frac{dn}{dT}\cdot \frac{\partial T}{\partial z} \end{array}\right.$$

### 2.2. Laser Atmospheric Subdivision Propagation Deflection Model

Based on the atmospheric refractive index, the laser deflection can be calculated for each isotropic air layer. Assuming that the angle of emission during propagation in the *i*-th layer is $\theta_{i}$, and refraction occurs at the boundary between the two layers at the time of incidence into the i+1 th layer, the angle of refraction is $\theta_{i+1}$, the deflection of the laser that occurs is $d\theta_{i}=\theta_{i+1}-\theta_{i}$. The refraction on the whole laser propagation can be expressed as Equation (7):

(7) $$d\theta \left(\lambda ,\theta_{1}\right)=\sum_{i=1}^{m}d\theta_{i}$$

In Equation (7), $\theta_{1}$ is the initial angle of laser incidence. Equation (4) can be obtained from the triangle sine formula and refraction theorem:

(8) $$N_{i}\sin\left(\theta_{i}\right)=N_{i+1}\sin\left(\theta_{i+1}\right)$$

**Figure 2:**
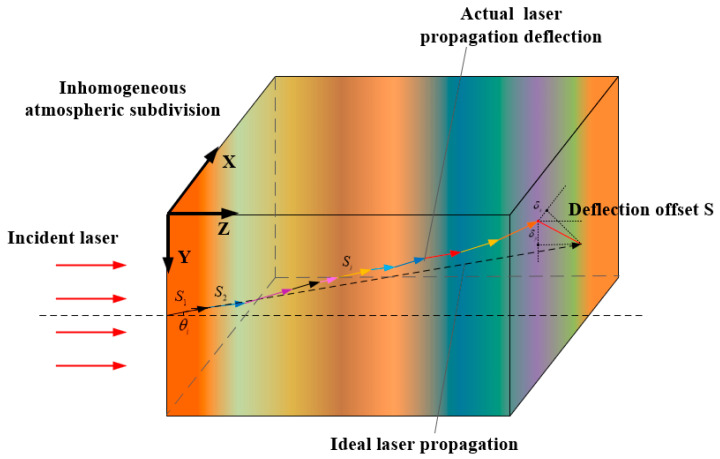
Schematic of the laser deflection through multiple inhomogeneous atmosphere layers.

There are two changes in the transmission path of a laser due to inhomogeneous atmospheric stratification: directional deviation and positional deviation. The accumulated laser deflection angle and offset after passing through multiple non-uniform air layers are shown in Figure 2. If the atmosphere through which the laser propagates is divided into an infinite number of non-uniform air layers, the laser can be considered to deflect at each layer boundary, while the refractive index within each layer remains uniform and isotropic.

Therefore, it can be assumed that the laser propagates in a straight line through each isotropic stratification, whereas in reality it propagates by an infinite number of infinitesimal arc lengths *ds*. Assuming that the initial outgoing direction vector of the light is $T_{0}\left(a_{0},b_{0},c_{0}\right)$, after many deflections by atmospheric disturbances, the detector incidence direction vector can be calculated as $T_{n}\left(a_{n},b_{n},c_{n}\right)$, which propagates along the *z*-axis, and the deflection angle of the laser spot can be calculated as shown in Equation (9).

(9) $$\cos\left(T_{0}^{\prime },T_{n}^{\prime }\right)=\frac{a_{0}\cdot a_{n}+c_{0}\cdot c_{n}}{\sqrt{a_{0}^{2}+c_{0}^{2}}+\sqrt{a_{n}^{2}+c_{n}^{2}}}$$

Expanding the deflection angle $\theta_{x},\theta_{y}$ in integral form as shown in Equation (10):

(10) $$\begin{array}{l} \theta_{x}=\int_{z_{1}}^{z_{2}}\frac{d^{2}x}{dz^{2}}dz=\frac{1}{n_{0}}{\int_{z_{1}}^{z_{2}}\left(\frac{\partial n}{\partial x}\right)}_{x,y,z}dz\\ \theta_{y}=\int_{z_{1}}^{z_{2}}\frac{d^{2}y}{dz^{2}}dz=\frac{1}{n_{0}}{\int_{z_{1}}^{z_{2}}\left(\frac{\partial n}{\partial y}\right)}_{x,y,z}dz \end{array}$$

In Equation (10), $n_{0}$ is the initial refractive index. The laser deflection displacement $\delta_{x}$ and $\delta_{y}$ in the x, y directions, respectively, can be calculated by Equation (11).

(11) $$\begin{array}{l} \delta_{x}=ds\int_{\varepsilon_{1}}^{\varepsilon_{2}}\frac{d^{2}x}{dz^{2}}dz=ds\int_{\varepsilon_{1}}^{\varepsilon_{2}}\frac{1}{n}\cdot \frac{\partial n}{\partial x}dz\\ \delta_{y}=ds\int_{\varepsilon_{1}}^{\varepsilon_{2}}\frac{d^{2}y}{dz^{2}}dz=ds\int_{\varepsilon_{1}}^{\varepsilon_{2}}\frac{1}{n}\cdot \frac{\partial n}{\partial y}dz \end{array}$$

Based on the measurement accuracy required of laser atmospheric transmission for the antenna subreflector pose measurement, the combination of Equations (1) to (11) shows that a temperature gradient of at least 1.39 K/m is necessary to achieve air layering and to analyze laser propagation deflection. Therefore, optimising the spatial distribution of environmental monitoring points and monitoring the high temperature gradient variations within the dual-reflector region is critical for enhancing the inversion accuracy of laser deflection.

### 2.3. Coupled Heat-Fluid Dynamics Model

To analyse the temperature gradient stratification more accurately, it is crucial to examine the effective temperature gradient of air thermal radiation acting on the main reflector and subreflector. Thermal radiation, which is electromagnetic radiation generated by the temperature of an object, is generally calculated using the Stefan–Boltzmann law, expressed as Equation (12).

(12) $$P=\varepsilon \sigma \left(T^{4}-T_{0}^{4}\right)$$

In Equation (12), *P* is the radiation power per unit area, ε is the emissivity, σ is the Stefan–Boltzmann constant, *T* is the surface temperature of the target, and *T*_0_ is the ambient temperature. According to the law of energy conservation, the radiant heat flow is equal to the heat flow by conduction through the air at steady state:

(13) $$\begin{array}{l} \rho c_{p}u\cdot \nabla T+\nabla \cdot q=Q+Q_{ted}\\ q=-k\nabla T=-k\frac{dT}{dz}\\ -n\cdot q=\varepsilon \sigma (T^{4}-T_{0}^{4})\cdot \left(1-F\right)+h(T-T_{0}) \end{array}$$

In Equation (13), **u** is the velocity vector, ∇T is the temperature gradient, ∇ is the divergence, q is the heat flux vector, *k* is the thermal conductivity, *Q* is the internal heat source, and $Q_{ted}$ is the external heat source, and n is the normal vector used to determine the direction of heat flux. −n⋅q denotes normal conductive heat flow, $\varepsilon \sigma (T^{4}-T_{0}^{4})$ denotes surface radiative heat dissipation, and *h* is the convective heat transfer coefficient. *F* is the geometric radiation angle coefficient; 1-*F* is the environmental radiation angle coefficient. *F* reflects the convergence ratio of radiant energy on the main reflector and subreflector, and its inclusion means the thermal radiation exchange between these surfaces is considered, which means the thermal radiation angular coefficient from the main reflector to the subreflector of the antenna. Based on this, the effective air-layer thickness ζ of the target to the air can be solved:

(14) $$\zeta =\frac{k\left(T-T_{0}\right)}{\varepsilon \sigma \left(T^{4}-T_{0}^{4}\right)\left(1-F\right)+h(T-T_{0})}$$

ζ represents the effective distance that the temperature falls from the surface temperature T to the ambient temperature $T_{0}$ when the surface dissipates heat by conduction and radiation under steady state conditions, which can be used as the theoretical basis for the air stratification between the antenna’s main reflector and subreflector. According to the condition of discontinuous rate of change in temperature gradient as the basis of air stratification, we assume that the boundary of air stratification is considered, and Equation (15) is satisfied.

(15) $$\;Boundary=\left\{\begin{array}{l} 1\;\;\;\;\;①\frac{\left|d^{2}T/dz^{2}\right|\cdot {\left(\Delta z\right)}^{2}}{std\left(T\right)}\geq \tau_{1}\;or\;\;②\frac{\left|dT/dz-\bar{\nabla }T\right|\cdot {\left(\Delta z\right)}^{2}}{std\left(\nabla T\right)}\geq \tau_{2}\;\\ 0\;\;\;\;\;\mathrm{otherwise} \end{array}\right.$$

**Figure 3:**
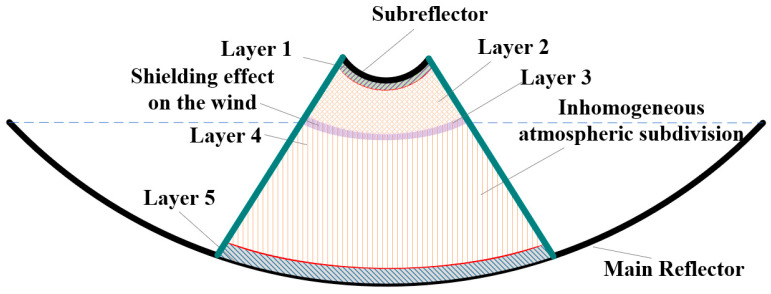
Schematic of the theoretical air layers between the large-aperture antenna’s main reflector and subreflector.

In Equation (15), Δz is the distance between two temperature measurement points, $std\left(T\right)$ and $std\left(\nabla T\right)$ are the temperature standard deviation and temperature gradient standard deviation respectively, $\bar{\nabla }T$ is the mean temperature gradient, $\tau_{1}$ and $\tau_{2}$ are the normalized curvature intensity threshold and temperature gradient deviation threshold respectively, which are determined based on experimental data statistics and laser deflection sensitivity analysis. The thresholds are set as $\tau_{1}=0.15$ (based on the 3σ criterion) and $\tau_{2}=1.39\;K/m$ (derived from the minimum resolvable laser deflection angle). These values effectively filter out random environmental thermal noise while preserving the distinct stratification boundaries. $d^{2}T/dz^{2}$ can identify mutation points in the temperature distribution, which depends on the local extremum, inflection point and gradient mutation. Combining these equations and the convergence effect of the main reflector and the thermal radiation effect of the main reflector and subreflector, the effective radiation thickness can be calculated, and the air layer excluding the thickness layer of the main reflector and the subreflector is the air layer with the same temperature as the ambient temperature based on the air boundary layer condition, and thus the air layer between the main reflector and the subreflector can be divided into three layers. However, due to the huge aperture of the main reflector, it has a shielding effect on the wind, so a more reasonable layering needs to be verified by simulation and experiment. In order to improve the accuracy of laser deflection measurement, the three layers of air layering are subdivided into five layers, as shown in Figure 3.

This paper adopts a discrete air segmentation method rather than a continuous integral refractive index gradient primarily because the experimental data acquisition is inherently discrete. Consequently, the discrete layering approach aligns more directly with the experimental measurements. In contrast, a continuous gradient would necessitate interpolation, introducing potential artificial smoothing. Furthermore, the proposed method focuses on characterizing the actual stratification phenomenon between the main reflector and subreflector. This approach provides practical guidance for optimizing environmental monitoring schemes in large-aperture antenna engineering.

## 3. Simulation and Experiment Verification

To analyze the deflection characteristics of laser atmospheric propagation within the complex environment of large-aperture antennas, high-precision monitoring of the flow field between the main reflector and subreflector is required. This necessitates internal non-uniform stratification modelling of the antenna. The antenna air stratification theory proposed in this paper must be validated through a combination of numerical simulation and experiment.

**Figure 4:**
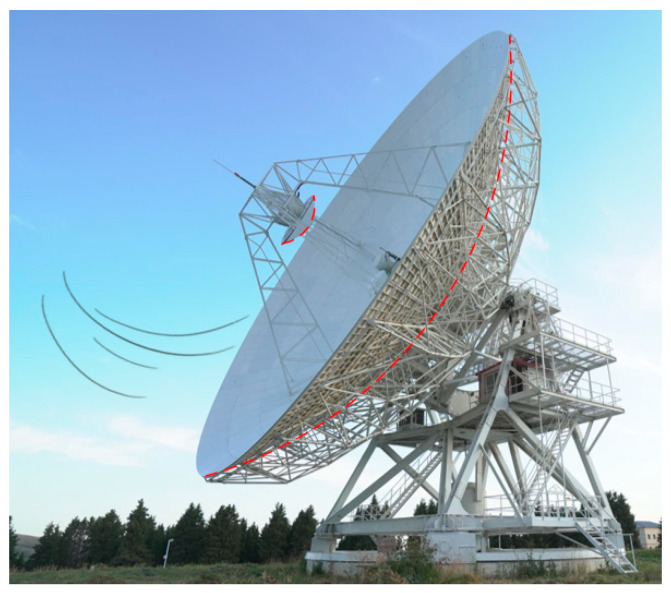
Nanshan 26m aperture and fully steerable radio telescope antenna.

Based on the CFD numerical simulation method and the stratification theory proposed in this paper, a two-dimensional simplified model is established for the Nanshan 26 m fully steerable radio telescope antenna (as shown in the red dashed outline in Figure 4). It is explicitly acknowledged that the airflow field around a large-aperture parabolic antenna is inherently three-dimensional. Compared to the turbulent external macro environment, the region between the main reflector and subreflector exhibits relatively homogeneous stratification. Regarding non-uniformities, localized minor turbulence is disregarded due to its negligible impact on laser refraction. Conversely, extreme or highly complex environmental conditions are excluded from the analysis, as high-precision measurement operations are inherently precluded under such scenarios. Although 3D modeling offers higher fidelity, its computational demands are substantial. Consequently, 2D simulation is used for temperature stratification analysis. This approach provides sufficient accuracy for the macroscopic assessment of large-aperture antenna environments while significantly optimizing computational resources.

This model focuses on the thermodynamic characteristics of the flow field between the main reflector and the subreflector. Considering solar radiation on the dual-reflector system and the thermal focusing effect of the main reflector, the temperatures of the subreflector, main reflector, and ambient air are set as t_1_, t_2_, and t_3_, respectively. Direct solar radiation causes the main reflector temperature t_2_ to be higher than the ambient air temperature t_3_. Owing to the structural characteristics of the dual-reflector system, thermal focusing leads to the subreflector temperature t_1_ being several tens of degrees higher than that of the main reflector. For verification purposes in the simulation, t_1_, t_2_, and t_3_ are set to 50 °C, 25 °C, and 10 °C, respectively. The curvature parameters of the Nanshan 26 m antenna’s main reflector and subreflector are retained in the simulation, with the left boundary set as a velocity inlet (3 m/s).

**Figure 5:**
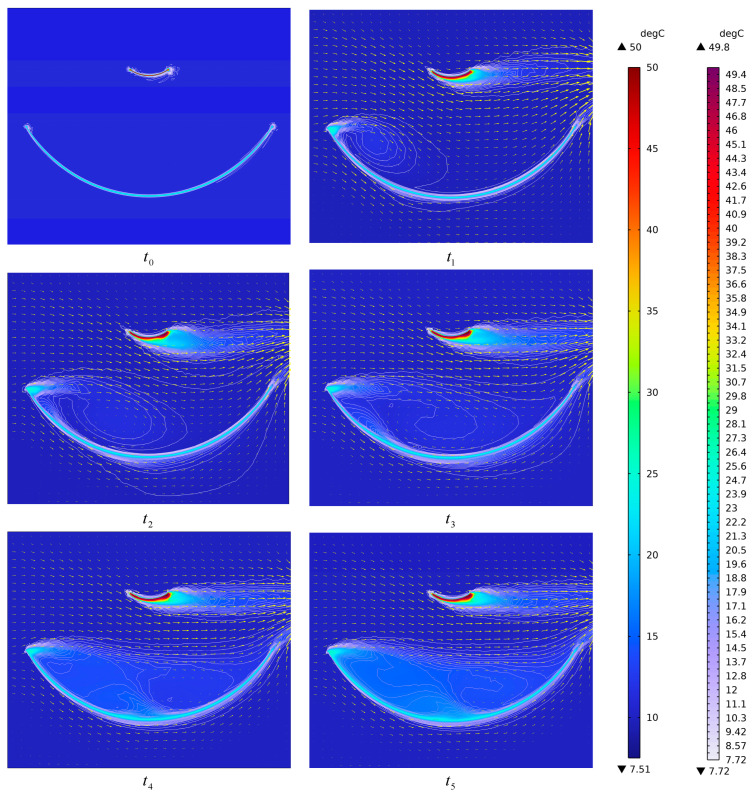
The simulation of the two-dimensional temperature distribution of the antenna at times $t_{0}$ to $t_{5}$.

Figure 5 shows the two-dimensional simulated temperature distribution of the antenna at the initial thermally balanced state (at time $t_{0}=0\;s$), as well as the simulated temperature and wind direction distributions at five subsequent times from t1 to t5 ($t_{1}=5\;s$, $t_{2}=10\;s$, $t_{3}=15\;s$, $t_{4}=50\;s$, and $t_{5}=100\;s$). It can be observed from Figure 5 that under thermal equilibrium ($t_{0}$) in the absence of forced convection from wind, only three air layers exist between the main reflector and the subreflector: the main reflector air layer, the subreflector air layer, and the ambient air layer. When the airflow is in a stable state, as illustrated in the simulation results at t4 and t5, the air between the two reflectors can be divided into five distinct air layers: the subreflector air layer, the ambient temperature layer, the forced convection layer, the eddy current ambient air layer, and the main reflector air layer. The differentiation of these layers is based on the stratification principle defined in Equation (15), where a distinct layer boundary is introduced when the local temperature gradient deviation exceeds the threshold or the curvature intensity exceeds.

**Figure 6:**
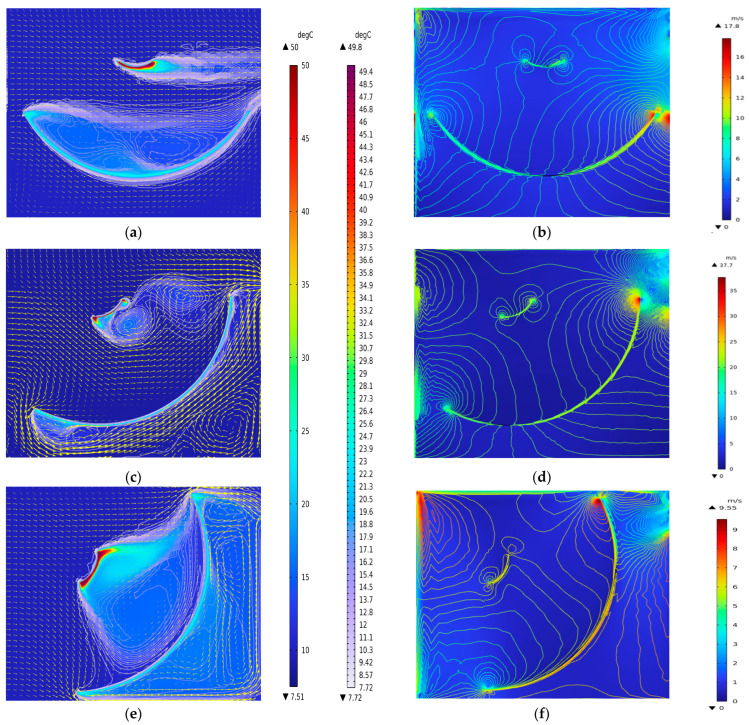
Simulation of wind speed, wind direction and temperature at different angles of rotation of the 2D antenna main reflector and subreflector. (**a**) 0° temperature field and wind direction simulation, (**b**) 0° velocity field simulation, (**c**) 30° temperature field and wind direction simulation, (**d**) 30° velocity field simulation, (**e**) 60° temperature field and wind direction simulation, (**f**) 60°velocity field simulation.

Figure 6 shows the simulated temperature and velocity fields of the antenna in the vertical state (0°) and at rotation angles of 30° and 60°, respectively. In the temperature simulations, the yellow arrows indicate wind direction, and the contour lines represent isotherms. In the velocity simulations, the contour lines correspond to velocity contours. In Figure 6a,c,e, the upper color bar represents the temperature field, while the lower color bar denotes the isotherm temperature values.

The simulation results in Figure 6 show that the subreflector of the radio telescope antenna exhibits the highest temperature, and its thermal convective dissipation maintains the surrounding air at a high temperature. When the antenna attitude changes, the flow fields near the main reflector and subreflector show significant temperature gradient stratification. By comparing the temperature and velocity field simulations of the antenna at three different angles, it can be observed that the environmental field is more complex when the antenna is at the 0° position. Assuming that the laser is emitted from the center of the main reflector toward the subreflector, the laser can be placed in a more stable environmental field for subreflector pose measurement when the antenna is oriented at 30° or 60°, thereby reducing the influence of the environmental field on measurement accuracy. Therefore, this paper focuses on analyzing the temperature gradient between the main reflector and subreflector when the antenna is at 0°.

**Figure 7:**
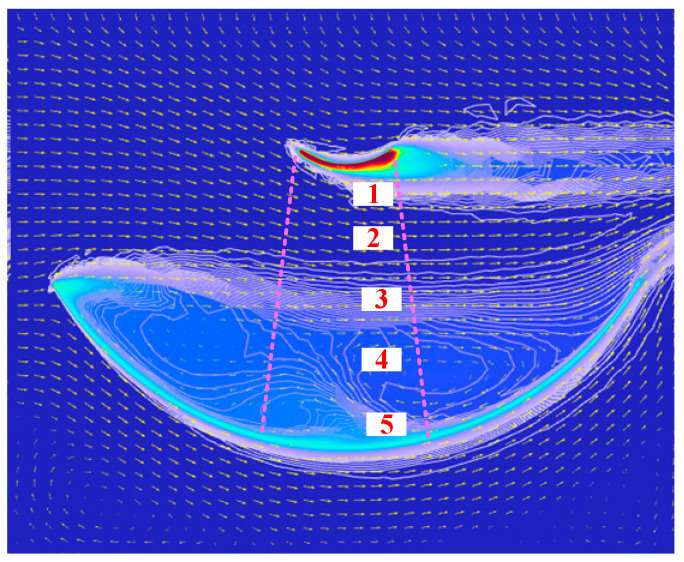
Air stratification between the antenna main reflector and subreflector. The pink dashed line represents the environmental monitoring area.

In the antenna 0°state, the unobstructed region between the main reflector and subreflector exhibits uniform flow velocity and direction. However, boundary layer effects near the main reflector generate localised vortices, inducing a heterogeneous temperature distribution. Based on the temperature gradient characteristics, the region between the main reflector and subreflector can be divided into five air layers, as shown in Figure 7, which validates and refines the atmospheric subdivision theory proposed in this paper. It should be noted that the height-dependent temperature variation model and thermal radiation model are not coupled in this simulation, which would lead to the existence of multiple homogeneous air stratifications within these five inhomogeneous stratifications, and thus will be considered during the actual antenna environment measurement experiments.

The non-uniform temperature gradient between the main reflector and subreflector of the large-aperture radio telescopes is analyzed theoretically and via simulation, based on the premise that significant temperature differences exist between these surfaces due to solar radiation. The impact of the temperature gradient on laser propagation deflection at different times lies in its variation, which depends on external environmental conditions such as season, cloudy or sunny weather. However, the change in temperature gradient is only influenced by wind flow at different times within the same stable environment.

## 4. Experiments and Results

**Figure 8:**
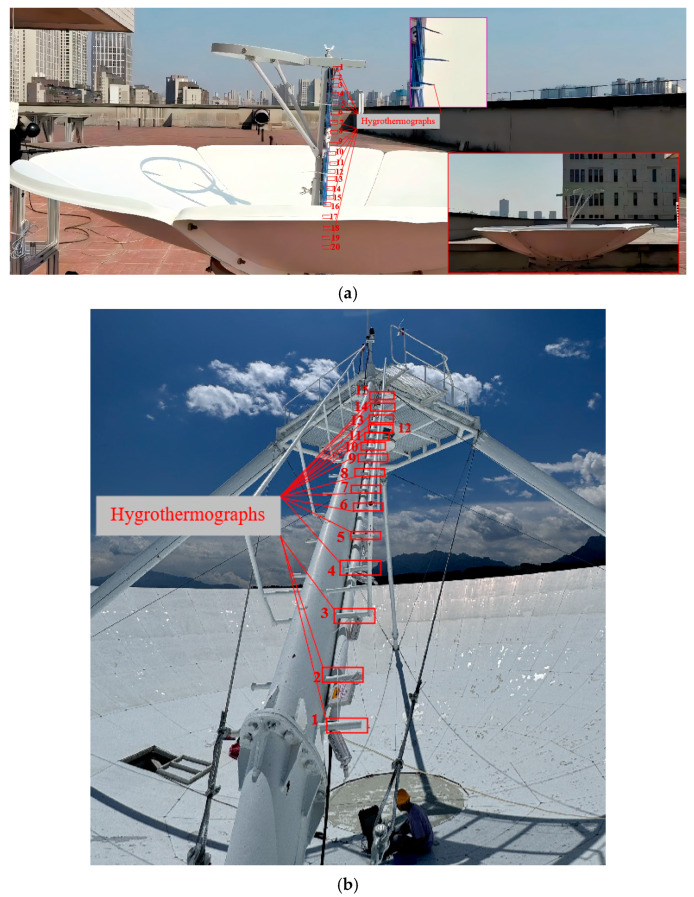
Environment monitoring experiments. (**a**) Environment monitoring experiments of the antenna model; (**b**) Environment monitoring experiments of the Nanshan 25 m antenna.

An environmental monitoring system was established to validate the proposed method through temperature measurement experiments conducted on both an antenna scale model and the actual Nanshan 25 m antenna. The environmental monitoring equipment consisted of hygrothermographs evenly distributed in the support structure between the main reflector and subreflector. The parameters of the hygrothermographs are as follows: temperature accuracy ±0.1 °C, humidity accuracy ±1.5% RH, temperature measurement range −40 °C to 85 °C, and humidity measurement range 0–100% RH. Figure 8 shows the environmental monitoring experiments for the antenna scale model and the Nanshan 25 m antenna, respectively.

**Figure 9:**
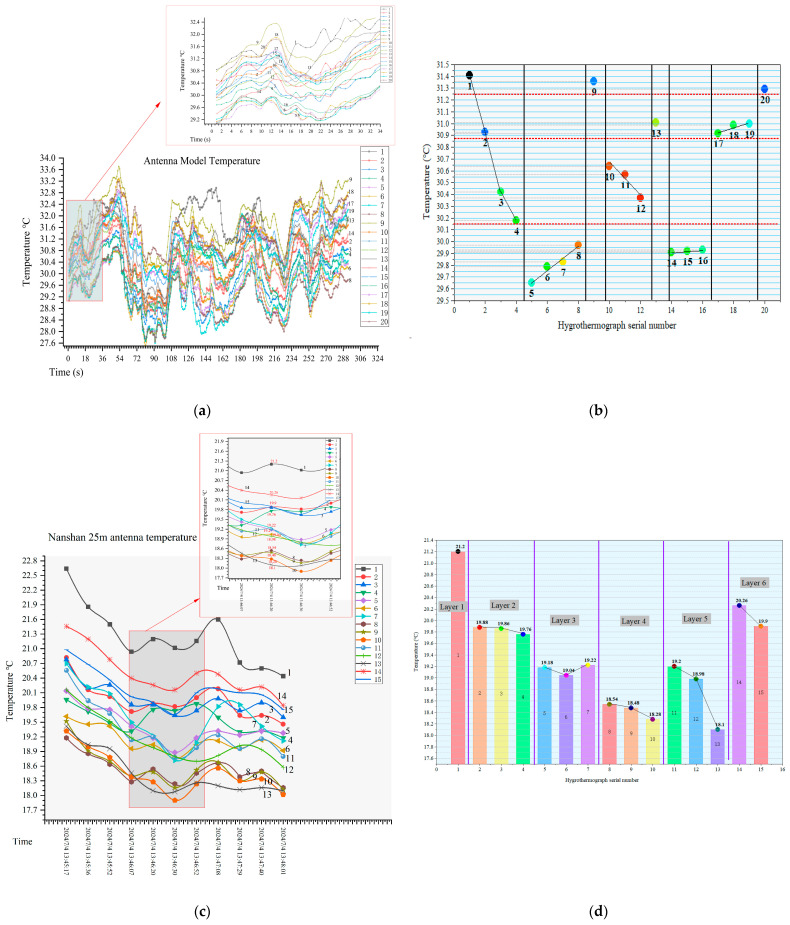
Antenna model temperature and Nanshan 25 m antenna temperature. (**a**) is the antenna model temperature, (**b**) is the antenna model temperature at a certain moment. (**c**) is the Nanshan 25 m antenna temperature, and (**d**) is the Nanshan 25 m antenna temperature at a certain moment.

The antenna model has a main reflector diameter of 1350 mm, with a distance of 600 mm between the main reflector and the subreflector. The hygrothermographs were placed at 30 mm intervals along the support structure. The unobstructed section of the support measures 340 mm, meaning sensors numbered 1 to 8 are located within the subreflector region and the open air zone. Figure 8 presents the experimental results. The temperature range recorded for the antenna model was 27.8 °C to 30.8 °C, while the ambient air temperature was 28 °C. As shown in Figure 8a and Figure 9a, sensors numbered 9 and 13 were exposed to direct sunlight; therefore, their data were excluded from the analysis. The region between the main reflector and subreflector of the antenna model can be divided into six distinct temperature gradient layers, with both reflectors showing elevated temperatures. Based on Figure 9b, it can be concluded that at a given time, sensors 1 to 4 correspond to temperature-gradient layers on the subreflector, sensors 5 to 8 represent layers in the unobstructed air, and sensors 17 to 20 correspond to gradient layers on the main reflector. The maximum temperature on the subreflector is higher than that on the main reflector. However, due to the relatively small overall size of the antenna model, the entire air layer is easily heated by solar radiation, which explains why the minimum temperature of the air layer remains above the ambient temperature.

The main reflector of the Nanshan 25 m antenna has a diameter of 25 m, and the distance between the main reflector and the subreflector is approximately 6 m. The hygrothermographs are evenly distributed along the support structure. Although the temperature sensors on the support frame may introduce measurement errors due to the frame’s own thermal effects, this paper primarily focuses on relative temperature trends. To minimize the interference, the sensor probe was positioned in the air away from the frame and oriented away from it to effectively shield against thermal radiation from the frame, and ensure that the measured data reflects ambient air temperature. Furthermore, the residual thermal effects from the support frame persist in the data; such systematic biases can be identified and eliminated during post-processing by analyzing the overall trend of the sensor measurements. If temperature data is affected by thermal reflection from the support frame, this paper can eliminate the bias by analyzing the trend of temperature sensor variations.

The ambient temperature range during the measurement of the 25 m antenna was 17.9 °C to 22.7 °C, with an ambient wind speed of 2.93 m/s. According to Figure 8b and Figure 9c, the air between the main and subreflectors of the antenna can be divided into six temperature gradient layers. Combined with Figure 9d, it can be concluded that at a specific moment, the air between the dual reflectors of the large aperture antenna exhibits clearly distinct stratification. Temperatures are higher within the layers adjacent to the main reflector and subreflector. Sensors 8 to 10 correspond to the unobstructed air layer and represent the ambient air temperature, while sensors 14 and 15 correspond to the air temperature stratification near the subreflector. Theoretically, due to the radiative characteristics of the subreflector, its temperature should be higher than that of the main reflector. However, as shown in Figure 8b, the significant gap between the support structure and the subreflector edge prevents the thermal air from the subreflector from fully reaching the measurement point of sensor 15. In addition to the air stratification of the main and secondary surfaces, which strictly adheres to the thermal radiation thickness stratification theory, the temperatures of the other positions have ups and downs, which indicates that they are influenced by local eddy currents, and this also provides an implementable data basis for the subsequent precise monitoring of the atmosphere between the main reflector and subreflector of the large-aperture antenna. It should be noted that the actual number of air layers depends on their effect on laser deflection: the number of layers decreases if the temperature gradient threshold increases, and increases if the threshold decreases.

**Figure 10:**
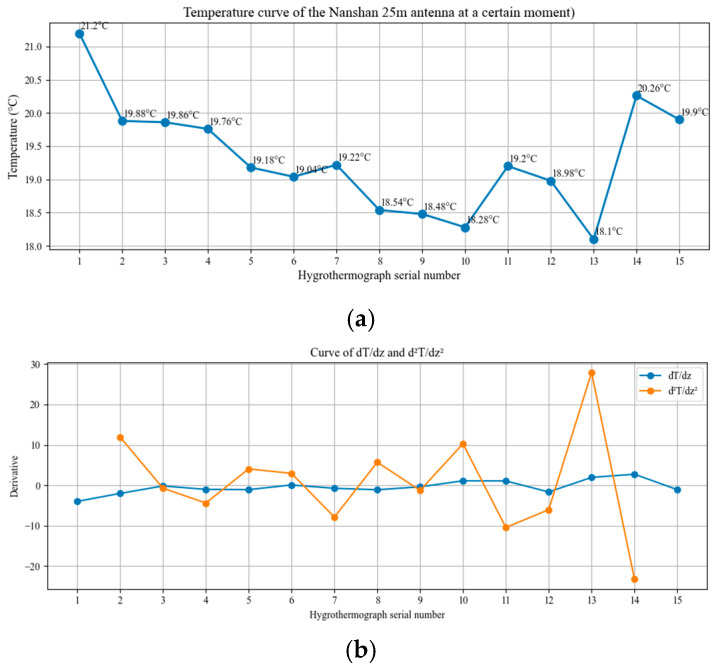
Temperature variation at a particular moment in Figure 9d. (**a**) is the temperature curve at a particular moment in Figure 9d, (**b**) is the temperature gradient and its rate of change at a particular moment in Figure 9d.

Figure 10 shows the rate of change curve of the temperature gradient $d^{2}T/dz^{2}$ and temperature gradient dT/dz at a particular moment in Figure 9d. According to the local extreme point and gradient mutation by Equation (15), the air stratification can be divided into 6 air layers according to the hygrothermograph’s serial number: 1, 2–4, 5–7, 8–10, 11–13, 14–15; this verifies the validity and feasibility of the proposed stratification theory. What is clear is that if the temperature gradient threshold increases, the air stratification can be divided into 5 air layers according to the hygrothermograph’s serial number: 1, 2–7, 8–10, 11–13, 14–15.

Applying the proposed stratification model to the experimental data (Figure 9) yields a theoretical laser spot deviation of approximately 0.011 mm over a 6 m propagation path, which serves as a conservative estimate given that the actual thermal gradients between the main reflector and subreflector are expected to be more severe than those measured at the support structure, leading to even larger deflections. Notably, this environmental deviation is superimposed onto the system’s intrinsic error of 0.0131 mm [40]. Since the atmospheric bias is comparable to the system’s precision limit, ignoring it would significantly degrade the laser measurement accuracy. Therefore, studying the stratification between the main reflector and subreflector of the large-aperture antenna plays a crucial role in improving the accuracy of the laser atmospheric propagation deflection.

## 5. Conclusions

This paper addresses the issue of laser measurement errors caused by harsh environmental conditions between the dual reflectors of the large-aperture antennas. A theory of inhomogeneous air stratification within the antenna is proposed, and a thermal-fluid coupled dynamic model of the dual-reflector system is established. The effectiveness and feasibility of this theory in characterizing asymmetric flow fields and temperature stratification were verified through numerical simulations and actual measurement experiments conducted on the Nanshan 25 m antenna. This study provides an innovative environmental modeling theory and experimental basis for high-precision laser-based antenna attitude measurement. Future work will integrate the temporal temperature difference method and environmental black-box modeling to refine the laser propagation deflection model further and enhance the accuracy of laser measurements.

## Data Availability

The data presented in this study are available on request from the corresponding author.
